# Effectivity of treatment for children with functional dyspepsia

**DOI:** 10.1038/s41598-022-05380-y

**Published:** 2022-01-27

**Authors:** Corinne Légeret, Yvonne Stienen, Raoul Furlano, Henrik Köhler

**Affiliations:** 1 University Children’s Hospital of Basel, Spitalstrasse, 4055 Basel, Switzerland; 2 Children’s Hospital of Aarau, Tellstrasse 9, 5001 Aarau, Switzerland

**Keywords:** Gastroenterology, Hepatology

## Abstract

Functional dyspepsia is very common in children of all ages and has a significant impact on the patient’s family and quality of life. Since the revision of the Rome IV criteria with the introduction of two subtypes, the prevalence of functional dyspepsia has increased, but currently no guidelines for the treatment are available. The aim of this study was to characterize patients, who have been diagnosed with functional dyspepsia and analyze the outcome of different treatments they received. This is a retrospective study of pediatric patients, diagnosed with functional dyspepsia between March 2017 and September 2020. All patients aged between 0 and18 years, who complained about gastric symptoms, have had a normal full blood count, a normal thyroid function, a negative coeliac screening, and most importantly normal macro- and microscopic findings on esophago-gastro-duodenoscopy were included in the study. Patient’s data were extracted from the medical record and three months after the performance of the endoscopy, parents were interviewed about the effect of the treatment. A total of 154 patients (66.2% female, 33.8% male) between the age of 4 and 18 years were included. In 113 (73.4%) the leading symptom was epigastric pain, followed by nausea (22; 14.3%) and a fifth of the patients (females: 18.6%; males: 21.2%) self-reported a current stressor in clinic. After receiving the diagnosis of a functional nature, families chose following treatments: 50 STW5 (32.3%, overall, 10.4% added dietary changes, alternative treatment, and psychology support), psychological support (22.7%), alternative treatments (e.g., hypnotherapy, meditation; 19.5%), dietary changes (12.9%), lifestyle changes (9.7%), no treatment (11%) and in 10.4% no treatment was needed as symptoms resolved after endoscopy had been performed. Only lifestyle changes (*p* = 0.03) in females, dietary changes (*p* = 0.035 for girls, *p* = 0.06 for boys) and STW5 in males (*p* = 0.043) showed a statistically relevant correlation regarding duration of symptoms. There was no correlation between location of symptoms and effectiveness of treatment. It is recommended to treat patients from both subgroups of functional dyspepsia differently, in accordance with the currently available explanatory models of underlying pathophysiological processes. In this cohort of patients this could not be verified. As all patients did benefit from any treatment, it is likely that the treatment itself was not accountable for the relief of symptoms, but that most patients benefit from education on the diagnosis, reassurance and a recommendation of a healthy lifestyle. Some patients might benefit from medications, small changes in the diet, psychological support or alternative treatment, but success depends on individual, unpredictable factor.

## Introduction

Functional gastrointestinal disorders (FGIDs) are very common in children of all ages. FGIDs is an umbrella term for a range of symptoms, which affect the gastrointestinal tract, but cannot be explained by structural or biochemical abnormalities^1^. FGIDs have a significant impact on the whole family, on health care related costs and mostly on the patient’s quality of life^2,3^.

There are no biochemical markers or structural abnormalities, which can be used to objectify the symptoms or monitor the progression of these disorders. In children, FGIDs are diagnosed according to the symptom-based Rome criteria, which have been revised in 2016 (Rome IV criteria^4^) and rely on the medical history and physical examination.

Dyspepsia refers to the Greek words ‘dys’ and ‘peptos’ and is translated with ‘hard to digest’. Functional dyspepsia (FD) is a sub-group of FGIDs, it is a clinical syndrome comprising chronic symptoms arising from the gastroduodenal region. According to the Rome criteria, the prototypical symptoms are postprandial fullness, early satiation, epigastric pain or burning not associated with defecation. The diagnosis of FD can be made, once one of those symptoms occur at least four times a month for at least 2 months and after appropriate evaluation, to ensure that the symptoms cannot be fully explained by another medical condition^4^. As the etiology of FD is multifactorial, including psychopathologic variables and gastric sensitivity^5^, and it often overlaps with other functional diseases^6^ there are no guidelines available for the treatment and different approaches like dietary manipulation, use of nutraceuticals or psychoeducation may be considered.

The purpose of our study was to characterize pediatric patients suffering from functional dyspepsia, analyze the outcome of treatment they received, in order to shed some light on the effectiveness of different approaches.

## Methods

This is a retrospective study of pediatric patients, who are under the care of the Children’s hospital Aarau and the University Children’s hospital of Basel, who have been diagnosed with functional dyspepsia between March 2017 and September 2020. All patients between the age of 0–18 years, who complained about gastric symptoms (pain, vomiting, nausea, fullness etc.) and had a normal full blood count, a normal thyroid function, a negative coeliac screening (normal t-transglutaminase, normal total IgA) and most importantly normal macro- and microscopic findings on esophago-gastro-duodenoscopy (all had biopsies taken from duodenum, stomach and esophagus) were included in the study. All patients with abnormal laboratory and/or histological findings, missing information or a refusal to participate (oral consent not given) were excluded.

Patient information was extracted from the medical record of each patient included in the study: Sex, date of birth, weight, height, symptoms (when several symptoms where mentioned, the leading one and a second symptom were noted), red flags, language spoken at home, school absence, underlying disease, risk factors (smoking, drugs, nutrition), family history, previous infection, stressors (asked as open ended question), previous treatment, duration of symptoms (more or less than 3 months), timepoint of appointment with a pediatric gastroenterologist, time between appointment in outpatient clinic and performance of endoscopy. After receiving the normal biopsy results of the upper endoscopy, which confirmed the functional nature of the symptoms, the patients and/or their families were informed about the etiology of functional dyspepsia. Patients were standardized offered a treatment with STW 5 (Iberogast®, an herbal mixture with statistically significant effects on patient’s epigastric functional symptoms found in double-blind and randomized studies^7^), or a referral to the psychologist for further support.

Three to four months after performance of endoscopy, all parents (to have a continuity throughout all patient ages, because parent’s reaction to the diagnosis is of interest and because we defined end of symptoms according to the parental statements when it stopped being talked about/they felt the child was not restricted by it anymore (in weeks after endoscopy)) were contacted by the same person by telephone and -after giving oral consent -were asked following questions: How is the child doing at the moment? Is the child missing school due to the symptoms? Is epigastric discomfort still an issue/is it still subject of discussion/is your child’s life restricted in any way by any symptoms? What kind of treatment did the family chose for the child? When did the discomfort/pain end being an issue (in weeks)? What did you think/feel when you learned about the functional nature of the problem of your child? Any thoughts/questions?

Red flags in the child’s history were defined as unintentional weight loss, dysphagia, repetitive vomiting and sanguineous vomit.

### Statistical analysis

Means with standard deviations (SD) were calculated for each of the measurements of interest. To identify correlations between qualitative data Pearson’s chi-squared test and Fisher’s exact test were performed. Data entry and statistical analysis were performed using R (version 4.0.3 (2020-10-10), R commander and XLSTAT (version 2020.5). A p-value of *p* < 0.05 was considered statistically significant.

### Ethical statement

The present study was approved by the local ethical committee (Ethics committee of Northwest Switzerland, EKNZ, Hebelstrasse 53, 4056 Basel, trial number 2015–322). On the follow-up phone call, informed consent was obtained from all parents. Furthermore, the study was conducted in accordance to the ethical principles laid down in the Declaration of Helsinki and its later amendments.

### Consent for publication

The study was approved by the local ethical committee.

## Results

A total of 154 patients (66.2% female, 33.8% male) between the age of 4 and 18 years were included in the study (see Table 1). Whilst obesity and ADHS were the most frequent underlying diseases in girls and in boys, males had significant (*p* = 0.03) more lifestyle risk factors.

**Table 1:** Patient’s characteristics.

	Female, n = 102	Male, n = 52
Mean age (range) in years	12.5 (4–18)	10.8 (4–17)
Mean height (range) in cm	148.9 (103.2–177.1)	145.7 (108–189)
Mean weight (range) in kg	46.8 (16–109)	41.9 (19.3–112.3)
Total number of patients, who speak one of the National languages at home, %	66/102, 64.7%	33/52, 63.5%
Underlying disease	17/102, 16.6%: 10 obesity 2 ADHS 1 bronchial asthma 1 atopic dermatitis 1 enuresis 1 scoliosis 1 functional constipation	11/52, 21.2%: 3 obesity 3 ADHS 1 bronchial asthma 1 global delayed development 1 hypothyroidism 1 pollen allergy 1 von Willebrand Syndrome Type I
Total patients with risk factors, percentage	5/102, 4.9%: 2 nicotine abuse 1 cannabis abuse 1 with previous eradication of H. pylori	6/52, 11.5%: 2 nicotine abuse 1 cannabis abuse 1 abouse of energy drinks (3–4 energy drinks/d) 2 patients, who only drink soft drinks (more than 1 L/day)
Total patients with a positive family history of a first degree relative with a gastrointestinal disease, percentage	21/102, 20.5%: 7 gastroesophageal reflux 5 gastritis 3 functional dyspepsia 3 inflammatory bowel disease (IBD) 1 eosinophilic esophagitis 1 H. pylori gastritis 1 coeliac disease	14/52, 26.9%: 4 H. pylori gastritis 3 gastroesophageal reflux 2 functional dyspepsia 2 irritable bowel syndrome 1 duodenal ulcer 1 Gallenstein 1 IBD

In 82/154 (53.3%) of all patient symptoms occurred less than 12 weeks before they were seen in a tertiary center by a pediatric gastroenterologist (Table 2). In 73.4% the leading symptom was epigastric pain, followed by nausea (14.3%) and a fifth of the patients (females: 18.6%; males: 21.2%) self-reported a current stressor in clinic.

**Table 2:** Description of the disease.

	Female, n = 102	Male, n = 52
Total number of patients with duration of symptoms less than 12 weeks, % Mean (range) in weeks	56/102, 54.9% 6.0 (0.3–12) weeks	26/52, 50% 6.3 (2–12) weeks
Total number of patients with duration of symptoms more than 12 weeks, % Mean (range) in months	46/102, 45.1% 12.5 (3–48) months	26/52, 50% 19.25 (3.5–48) months
Total number of patients with regular school absence, %	28/102, 27.4%	15/52, 28.8%
Main symptom, total (%)	78 (76.5%) epigastric pain 15 (14.7%) nausea 3 vomiting 3 burning pain 1 dysphagia 1 fullness 1 belching	35 (67.3%) epigastric pain 7 (13.5%) nausea 4 belching 3 vomiting 1 burning pain 1 dysphagia 1 fullness
Secondary symptom, total (%)	27/102, 26.5%: 9 nausea 4 belching 4 epigastric pain 2 fullness 2 fatigue 2 burning pain 1 loss of appetite 1 bloating 1 vomiting 1 hyperventilation	22/52, 42.3%: 6 vomiting 5 nausea 3 belching 2 epigastric pain 1 burning pain 1 dysphagia 1 fatigue 1 fullness 1 dizziness 1 halitosis
Total number of patients, who had a gastrointestinal infection before onset of symptoms, %	10/102, 9.8%	2/52, 3.8%
Total number of patients with self-reported stressors, %	19/102, 18.6%	11/52, 21.2%
Total number of patients, who received a medical treatment from the general pediatrician, %	68/102, 66.6%: 65 had PPI 3 had Iberogast	32/52, 61.5%: 31 had PPI 1 had antibiotics (Axomixicilline/Clarithromycine)
Total number of patients with red flags, %	8/102, 7.8%: 4 vomiting 1 dysphagia 3 unintentional weight loss	12/52, 23%: 9 vomiting 2 dysphagia 1 unintentional weight loss
Mean time (range) between clinic and endoscopy in days	16.4 (1–61)	16.1 (2–61)

Roughly a third (females: 27.4%, males: 28.8%) of all patients had a regular school absence, which did not correlate with the length of treatment needed until symptoms resolved.

Male patients had statistically significant (*p* = 0.03) more red flags in their history, than girls. After receiving the diagnosis based on a functional nature, families chose the following treatments: STW5 (in total 32.3%, overall, 10.4% added dietary changes, alternative treatment and psychology support), psychological support (22.7%), alternative treatments (19.5%), dietary changes (12.9%), lifestyle changes (9.7%), no treatment (11%) and in 10.4% no treatment was needed as symptoms resolved after endoscopy (see Table 3). Only lifestyle and diet changes (*p* = 0.03) in females, dietary changes (*p* = 0.035 for girls, *p* = 0.06 for boys) and STW5 in males (*p* = 0.043) showed a statistically relevant correlation in regard to duration of symptoms. In female patients, symptoms declined on average after 3.2 weeks, in male patients after 2.6 weeks. There was no significant correlation between the duration of the symptoms and the need of a psychological inpatient treatment, but all either had an underlying disease (ADHS, enuresis), a risk factor or a red flag in their personal history, only 42.8% reported a stressor. In 8 patients, symptoms were not resolved by the time of the follow-up or they were treated as an inpatient for symptoms, which might be associated with the underlying functional disease (school absence, somatization disorder).

**Table 3:** Treatment.

Total patients, who received treatment, % Duration of symptoms in weeks (range) until symptoms resolved after the endoscopy	Female, n = 102	Male, n = 52
STW5	35/102, 34.3%: 3.5 (1–12) weeks *p* = 0.06	15/52, 28.8%: 2.7 (0.6) weeks *p* = 0.043
Psychological support	25/102, 24.5%: 10 outpatient setting 4.2 (1–12) weeks *p* = 0.07 15 inpatient setting 4 (1–16) weeks	10/52, 19.2%: 6 outpatient setting 3.5 (1–6) weeks *p* = 0.08 4 inpatient setting (unknown duration, were partly still inpatient)
Alternative treatment	21/102, 20.6%: 7 osteopathy 3 hypnotherapy 3 breathing exercise 2 relaxation exercises 2 homeopathy 1 acupuncture 1 meditation 1 reflexology 1 anti-nausea wristbsand 4.8 (2–16) weeks *p* = 0.2	9/52, 17.3%: 3 breathing exercise 2 relaxation exercise 2 osteopathy 1 acupuncture 1 hypnotherapy 5.25 (2–15) weeks *p* = 0.6
Dietary changes	16/102, 15.7%: 4 went lactose free 3 reduced intake of gluten 1 no apple puree 1 ate more rice and potato 1 reduced fat 1 reduced nuts 1 reduced chocolate 1 reduced sugar 1 switched to water without gas 1 avoids tomatoes & oranges 1 avoids tomatoes, onions and garlic 2.4 (1–4) weeks *p* = 0.035	4/52, 7.7%: 2 went lactose free 1 added one apple tea a day Cow’s milk was replaced with oat milk 2.6 (0–4) weeks *p* = 0.05
Lifestyle changes (sleep rhythm, nicotine, energy drinks, weight reduction)	8/102, 7.8%: 1.5 (0–6) weeks *p* = 0.03	7/52, 13.5%: 2.8 (1–6) weeks *p* = 0.06
Number of Patients, who wished no treatment as symptoms completely resolved after the endoscopy	7/102, 6.8%: 0.0 (0–0)	9/52; 17.3% 0.0 (0–0)
Refusing treatment but wanted to observe natural course	11/102, 10.8%: 2.8 (0–8) weeks *p* = 0.045	6/52, 11.5%: 1.0 (0–3) weeks *p* = 0.039
Mean weeks (range) until symptoms declined in all patients	3.2 (0–16) weeks	2.6 (0–15) weeks
Reaction of parents	35.9% relieved, felt secure after scope 25.5% surprised/astonished 14.7% felt reassured, had presentiment 8.8% are disappointed, fear	40.4% relieved 26.9% surprised/astonished 11.5% felt reassured, had presentiment 1.9% disappointed

## Discussion

In our cohort of patients, we found a clear female dominance, which is in line with other studies regarding functional epigastric pain, where 10% of the population was found to fulfill the criteria of FD with a higher prevalence in women^8^. Studies in children show a similar prevalence^9^.

Pathophysiological features of FD are diverse, complex, not fully understood but seem to include psychological distress, particularly anxiety, slow gastric emptying, fundic disaccomodation, gastric hypersensitivity and mild immune activation—the role of the microbiome remains unclear at this point^10^. Our study confirms the findings of the current literature, in regard to risk factors and underlying diseases in FD: A hypothesis is, that FD may arise after an enteric infection through an inflammation of the small intestine or stomach^11^—7.8% of our patients indeed have had an infection of the gastrointestinal tract before the symptoms became chronic. 7.1% of the children had risk factors in their history (nicotine abuse and consumption of energy drinks), which are associated with FD^12^, whilst 19.4% self-reported a current stressor in their life. Perceived stress has been suggested to affect the symptom severity rather than the development of the symptoms itself^13^. It is repeatedly discussed whether being a carrier for H. pylori is a risk factor for triggering functional dyspepsia^11^—this can be ruled out in our cohort of patients, as we only included patients without any abnormal histopathological findings and therefore children with H. pylori or a corresponding gastric reaction were not included in this study. 11.7% of our patients were obese or had an underlying diagnosis of ADHS, both are clearly associated with FGDs^14,15^.

In 62.3% of all cases the pediatrician prescribed an acid suppression therapy (PPI) and referred the patient to the pediatric gastroenterologist after the anticipated effect not being achieved. Many gastroenterological societies^16^ advocate this strategy for treating dyspepsia in adult patients, as patients with dyspepsia, who experience reflux-type symptoms, rather than dysmotility issues or nausea, primarily benefit from this treatment. In 2016 the new Rome IV Criteria on pediatric gastrointestinal functional disorders were published^4^. With this new definition, FD came with two subtypes: Epigastric pain syndrome and postprandial distress syndrome. According to this new classification, 75.9% of the patients fulfilled the criteria for the epigastric pain syndrome.

It was striking that in half of all patients the duration of symptoms was less than 12 weeks before they were seen by a pediatric gastroenterologist and the mean time between consultation and the performance of an endoscopy was only 16 days. This is not due to urgent indications as only 12.9% of the patients had red flags, but it may reflect the easy access to a specialist’s care in Switzerland. A fraction underwent an endoscopy within less than 8 weeks since onset of symptoms, and formally did not fulfill the criteria of FD according to Rome IV criteria^4^ at this point. On one hand a swift endoscopy can be beneficial for patients, in the sense that they are quickly provided with a diagnosis which is concomitant with reassurance, on the other hand they have to undergo an invasive procedure. Interestingly, in our cohort there was no statistically significant correlation between the previous duration of symptoms and the time after the endoscopy, until they completely resolved. Also striking, is the prevalence of the different symptoms in our FD patients: Whilst postprandial fullness and early satiety is commonly described in those patients^17^, this was not verified in our cohort, where by far most patients suffered from epigastric pain. This fact is probably caused by a study bias. Severe epigastric pain causes not only fear in those patients, but the clinician might perform an endoscopy quicker in those patients, compared to the ones suffering mainly from early satiety- a classic functional symptom. In our study only patients were included, who had an endoscopy performed, which means we might have a bias in the composition of the cohort.

Since the etiology of FD is multifactorial (genetics, environment, stressors, coping strategies, social support, gastric sensation/motility/inflammation etc.), the corresponding treatment is as well and involves different approaches: psychotherapy, dietary changes, relaxation and stress management, PPI, herbal medicines, prokinetic drugs and laxatives. An herbal combination medicine STW 5 (Iberogast ®; Steigerwald Arzneimittelwerk GmbH, Germany) was developed to address overlapping and different symptoms in FD and has been used in central Europe since over 5 decades. Studies show that the herbal solution increases proximal gastric volume and antral motility^18^. Double-blind and randomized studies found statistically significant effects on patient’s symptoms with a comparable efficacy to a standard prokinetic, whilst STW 5 has a favorable tolerability, which is relevant for long-term treatment^7^. In our cohort, patients responded well to the treatment with STW5, but was statistically relevant in boys only. A fraction of the families added dietary changes to the treatment with STW5, which prevents an allocation of a direct therapeutic effect to the interventions.

A systematic meta-analysis of over 6400 studies found high fat food to be the major player in FD^19^. This phenomenon is most likely explained by the fat specific effect on the release of cholecystokinin (CCK) as well. This theory is undermined by studies, who overturn the inhibition of gastric motility with the administration of a CCD receptor antagonist after the ingestion of a high-fat meal^20^. Hence the recommendation for a healthy, balanced diet and regular meals. Another analysis of the literature shows that the most common foods, recognized by adult patients suffering from FD, appear to contain high concentrations of fermentable oligosaccharides, disaccharides, monosaccharides and polyols (FODMAPs^21^), which are widely accepted to be potential triggers for gastrointestinal symptoms. They can cause an increased intestinal fermentation and luminal water retention, both effects enhance abdominal distension and pain in patients with an altered visceral sensitivity^22^. Many patients recognize meals as triggering factors and lean on dietary manipulations as first-line management strategy. Unfortunately, a lot of articles are available with not validated nutritional advices combined with the lack of standardized dietary guidelines for FD, patients often tend to self-diagnose, empiric approaches are chosen and lead to the perception of ‘being intolerant’ to food^23^. We hypothesize to observe this phenomenon in our cohort of patients as well: Patients were not offered dietary changes, but families did it on their own without any medical support/recommendation. There was a statistically significant therapeutic effect in the patients with a self-restrictive diet, the benefit of the avoidance of some foods/nutrients such as lactose and fat can be explained by above mentioned processes. Whilst the consumption of rice was—as in our patient cohort—shown to be well tolerated by patients suffering from FD^24^, gluten-rich food seems to lead to a symptom-onset in FD patients by a decreased claudin-1 expression and mucosal immune activation^25^. Whilst some of the patients naturally made changes to their daily diet, where data support a corresponding benefit and a pathophysiologic explanation is available as they chose to start a low FODMAP diet (lactose free, reduction of gluten, onions and garlic), other families simply adapted a healthier diet (reduction of sugar and chocolate, water without gas) and others chose a path (adding apple tea, reduction of nuts, tomatoes), where no medical explanation for the success exists. Clearly, the number of patients is too small to make a statement regarding effectiveness of different diets. Authors caution about the increasing amount of these improvised and not controlled elimination diets, which can be unbalanced and could enhance anxiety toward that food, increasing visceral hyperalgesia and contributing to symptoms anticipation^26^.

Patients, who were advised to foster health-conductive behaviors (abstaining use of tobacco and cannabis, increase physical activity, intake of regular meals etc.) were reported to have a statistically significant quick delice of symptoms after endoscopy. Naturally, those patients did not initiate and maintain healthy behaviors within this short amount of time, but presumably they did not have enough self-determined motivation to do so^27^. As parents tend to push for a healthier lifestyle, assumably they did not dare to complain about the symptoms in order to prevent hearing they should address the underlying problem.

21.4% of our patients had no symptoms anymore right after the endoscopy or did not want to start a treatment. Studies show that patients with a higher pain score before an upper endoscopy identify as predictors for higher pain score after pediatric esophagogastroduodenoscopies^28^, which might either indicate that the pain in our patients was not severe in the beginning, or the diagnosis of a functional disease was enough to provide the patient with security.

In patients, who chose a psychological support or tried an alternative treatment, duration until the symptoms declined was slightly longer, however effective. The aim is clearly to resolve stressor and/ or provide the child with relaxation procedures. Since the follow-up was three months after endoscopy, it is unknown, which treatment was the most effective one, on the long-term.

Unfortunately, we did not identify one single risk factor in the history/symptoms for patients, whose symptoms were so severe that an intense treatment as an inpatient was needed, but all persons concerned either had a psychological underlying diagnosis (ADHS, enuresis), a risk factor or a red flag in their history, but only 42.8% named a stressor.

An international pediatric committee published a systematic review in 2018^29^ where no evidence was found to support the use of pharmacological drugs. They recommend treating each subtype based on adult data, as more data become available. For the epigastric pain syndrome subtype, the use of PPI as first line treatment is recommended. 57.9% of our patients classify as suffering from epigastric pain syndrome and 65% of them were treated with PPI without success. For the postprandial distress syndrome subtype, the committee recommends the use of fundal relaxant medications. In our cohort this equals the treatment with STW5, which did not have a better effect than in children with epigastric pain syndrome. Dietary changes impair motility (via CCK) or intestinal dilation and therefore patients with postprandial distress should benefit from those therapeutic approaches, which was not shown in our cohort.

A clear limitation of this study is its retrospective nature: In this case it comes with a lack of standardized questionnaire in the first consultation regarding symptoms and stressors. Although the same three consultants lead the clinics and asked similar questions (all open-end), it was not a standardized setting. This issue was tried to be solved by including only patients in the study, who answered all the questions. As the inclusion criteria are strict, the cohort of participants is small, which is another limitation to this study. It led to small sub-groups of patients with different leading symptoms, which chose a certain treatment, therefore it’s statistical significance is small, but might only show a trend. It is difficult to measure and therefore compare and observe the course of functional symptoms, especially in a retrospective study. We therefore chose to have the follow-up interview with the parents, and defined resolution of the symptoms as the point, where parents felt that their child’s quality of life was not restricted anymore.

Only 13.6% of all parents assumed that their child’s symptoms could be of functional nature, while most of the parents were relieved, surprised/astonished and some were even disappointed after hearing that ‘nothing was found on biopsies’. All those emotional reactions show that a somatic disease was clearly expected by them, although families were informed about possible differential diagnosis (including FD) before the endoscopy. This is in line with the literature^30^, where it was shown that pediatric patients suffering from FD and their parents are more inclined to see a monocausal and most frequently a physical explanation, than to seek a multicausal biopsychosocial explanation.

## Conclusion

With the renewal of the Rome criteria and the introduction of the two subgroups in 2016, the prevalence of functional dyspepsia suddenly exceeds the prevalence of IBS^9^. It was previously the most prevalent functional abdominal pain disorder in children^31^, therefore most literature was focused on this diagnosis and treatment and little attention was being paid to functional dyspepsia, which was assumed to be uncommon. With the new definition, patients, who might have classified as IBS previously, can now fulfill the criteria for FD, subgroup postprandial distress syndrome. Although this is currently the largest group of pediatric patients with functional gastrointestinal pain, currently no guidelines are available for its treatment.

Looking at our cohort of 154 patients, families tend to choose their own treatment, which in fact, were all successful, some quicker than others. Whilst analyzing available explanatory models for pathophysiological processes and their recommended treatment, it was striking that in our cohort of patients it did not add up. Lifestyle changes and PPI should—from a pathophysiological point of view—improve epigastric pain syndrome, whereas STW5 and dietary changes should relieve postprandial distress syndrome. In our patients this could not be verified: Patients from both subgroups did benefit from any treatment they chose. As current explanatory models of the treatment cannot be transferred into our cohort of patients, a strong suspicion emerges, that the treatment chosen is not accountable for the relief of the symptoms. Ultimately this leads to the recommendations as given decades ago, to educate families on the diagnosis, provide reassurance and advocate a healthy lifestyle. Some patients might benefit from medications, small changes in the diet, psychological support or alternative treatment, but success depends on individual, unpredictable factors. Additional treatments can give the child the chance to save his face when returning home or back to school.

## Data Availability

The dataset used/analysed is available from the corresponding author on reasonable request.

## Abbreviations

ADHS: Attention deficit hyperactivity disorder
FD: Functional dyspepsia
FGIDs: Functional gastrointestinal disorders
IBS: Irritable bowel syndrome
PPI: Proton pump inhibitors
STW5: Iberogast®

## References

[1] Hyams JS, Di Lorenzo C, Saps M (2016). Functional disorders: Children and adolescents. Gastroenterology.

[2] Devanarayana NM, Rajindrajith S, Benninga MA (2014). Quality of life and health care consultation in 13 to 18 year olds with abdominal pain predominant functional gastrointestinal diseases. BMC Gastroenterol..

[3] Varni JW, Bendo CB, Nurko S (2015). Health-related quality of life in pediatric patients with functional and organic gastrointestinal diseases. J. Pediatr..

[4] https://theromefoundation.org/rome-iv/rome-iv-criteria/

[5] Perez ME, Youssef NN (2007). Dyspepsia in childhood and adolescence: Insights and treatment considerations. Curr. Gastroenterol. Rep..

[6] Friesen CA, Rosen JM, Schurman JV (2016). Prevalence of overlap syndromes and symptoms in pediatric functional dyspepsia. BMC Gastroenterol..

[7] Ottillinger B, Storr M, Malfertheiner P, Allescher HD (2013). STW 5 (Iberogast®)—A safe and effective standard in the treatment of functional gastrointestinal disorders. Wien Med. Wochenschr..

[8] Koloski NA, Jones M, Talley NJ (2016). Evidence that independent gut-to-brain and brain-to-gut pathways operate in the irritable bowel syndrome and functional dyspepsia: A 1-year population-based prospective study. Aliment. Pharmacol. Ther..

[9] Saps M, Velasco-Benitez CA, Langshaw AH, Ramírez-Hernández CR (2018). Prevalence of functional gastrointestinal disorders in children and adolescents: Comparison between Rome III and Rome IV criteria. J. Pediatr..

[10] Talley N, Ford A (2015). Functional dyspepsia. N. Engl. J. Med..

[11] Du LJ, Chen BR, Kim JJ, Kim S, Shen JH, Dai N (2016). Helicobacter pylori eradication therapy for functional dyspepsia: Systematic review and meta-analysis. World J. Gastroenterol..

[12] Jaber N, Oudah M, Muttappallymyalil J (2016). Dietary and lifestyle factors associated with dyspepsia among preclinical medial students in Ajman. Central Asian J. Glob. Health.

[13] Van Oudenhove L, Vandenberghe J, Fischler B (2008). Determinants of symptoms in functional dyspepsia: Gastric sensorimotor function, psychosocial factors or somatization?. Gut.

[14] Galai T, Moran-Lev H, Cohen S (2020). Higher prevalence of obesity among children with functional abdominal pain disorders. BMC Pediatr..

[15] Kedem S, Yust-Katz S, Carter D (2020). Attention deficit hyperactivity disorder and gastrointestinal morbidity in a large cohort of young adults. World J. Gastroenterol..

[16] Moayyedi PM, Lacy BE, Andrews CN, Vakil N (2017). ACG and CAG clinical guideline: Management of dyspepsia. Am. J. Gastroenterol..

[17] Shava U, Srivastava A, Mathias A, Kumar N, Yachha SK, Gambhir S, Poddar U (2021). Functional dyspepsia in children: A study of pathophysiological factors. J. Gastroenterol. Hepatol..

[18] Pilichiewicz A, Horowitz M, Feinle-Bisset C (2007). Effects of Iberogast on proximal gastric volume, antropyloroduodenal motility and gastric emptying in healthy men. Am. J. Gastroenterol..

[19] Duncanson KR, Talley NJ, Walker MM, Burrows TL (2018). Food and functional dyspepsia: A systematic review. J. Hum. Nutr. Diet..

[20] Feinle C, Meier O, Otto B, D'Amato M, Fried M (2001). Role of duodenal lipid and cholecystokinin A receptors in the pathophysiology of functional dyspepsia. Gut.

[21] Duncanson KR, Talley NJ, Walker MM, Burrows TL (2018). Food and functional dyspepsia: A systematic review. J. Hum. Nutr. Diet..

[22] Eswaran S, Farida JP, Green J, Miller JD, Chey WD (2017). Nutrition in the management of gastrointestinal diseases and disorders: The evidence for the low FODMAP diet. Curr. Opin. Pharmacol..

[23] Lee IS, Preissl H, Giel K, Schag K, Enck P (2018). Attentional and physiological processing of food images in functional dyspepsia patients: A pilot study. Sci. Rep..

[24] Miwa H, Ghoshal UC, Bak YT (2012). Asian consensus report on functional dyspepsia. J. Gastroenterol. Hepatol..

[25] Du L, Shen J, Kim JJ, He H, Chen B, Dai N (2018). Impact of gluten consumption in patients with functional dyspepsia: A case-control study. J. Gastroenterol. Hepatol..

[26] Pesce M, Cargiolli M, Cassarano S (2020). Diet and functional dyspepsia: Clinical correlates and therapeutic perspectives. World J. Gastroenterol..

[27] Ntoumanis N, Lonsdale C, Williams GC (2021). A meta-analysis of self-determination theory-informed intervention studies in the health domain: Effects on motivation, health behavior, physical, and psychological health. Health Psychol. Rev..

[28] Galai T, Yerushalmy-Feler A, Heller NP (2020). Age and pain score before gastrointestinal endoscopies in children are predictors for post procedure pain. BMC Gastroenterol..

[29] Browne PD, Nagelkerke SCJ, van Etten-Jamaludin FS, Benninga MA, Tabbers MM (2018). Pharmacological treatments for functional nausea and functional dyspepsia in children: A systematic review. Expert. Rev. Clin. Pharmacol..

[30] Hulgaard DR, Rask CU, Risor MB, Dehlholm G (2020). Illness perceptions of youths with functional disorders and their parents: An interpretative phenomenological analysis study. Clin. Child Psychol. Psychiatry.

[31] Korterink JJ, Diederen K, Benninga MA, Tabbers MM (2015). Epidemiology of pediatric functional abdominal pain disorders: A meta-analysis. PLoS ONE.

